# Glycomacropeptide-Based Protein Substitutes for Children with Phenylketonuria in Italy: A Nutritional Comparison

**DOI:** 10.3390/nu16070956

**Published:** 2024-03-27

**Authors:** Martina Tosi, Laura Fiori, Veronica Maria Tagi, Mirko Gambino, Chiara Montanari, Alessandra Bosetti, Gianvincenzo Zuccotti, Elvira Verduci

**Affiliations:** 1 Department of Pediatrics, Vittore Buzzi Children’s Hospital, University of Milan, 20154 Milan, Italy; martina.tosi@unimi.it (M.T.); laura.fiori@asst-fbf-sacco.it (L.F.); veronica.tagi@unimi.it (V.M.T.); mirko.gambino@unimi.it (M.G.); chiara.montanari@unimi.it (C.M.); alessandra.bosetti@asst-fbf-sacco.it (A.B.); gianvincenzo.zuccotti@unimi.it (G.Z.); 2 Department of Health Sciences, University of Milan, 20146 Milan, Italy; 3 Department of Biomedical and Clinical Science, University of Milan, 20157 Milan, Italy; 4 Metabolic Diseases Unit, Department of Pediatrics, Vittore Buzzi Children’s Hospital, University of Milan, 20154 Milan, Italy

**Keywords:** phenylketonuria, phenylalanine, protein substitutes, glycomacropeptide, diet, non-communicable diseases

## Abstract

Advancements in food science technology have allowed the development of new products for the therapeutic management of inherited metabolic diseases such as phenylketonuria (PKU). Glycomacropeptide (GMP), a peptide derived from casein, is naturally low in phenylalanine (Phe) and, thus, adequate for protein substitutes (PSs) for the management of PKU in children. This review aims primarily to analyse the differences in the nutritional composition of GMP-based protein substitutes in different formulations (ready to drink, powdered, and bars), and secondarily to assess the quality of these products, comparing their nutritional composition with that of standard amino acid (L-AA) mixtures. Thirty-five GMP-based PSs produced by six different companies were included in this review: twenty-one powdered PSs, eight ready to drink, and six bars. The analysis revealed great heterogeneity not only among the different formulations (powdered, ready to drink, and bars) but also within the same group, in terms of energy content and nutritional composition. GMP-based PSs were shown to have higher contents of sugars and saturated fatty acids compared to L-AA PSs, especially in ready-to-drink formulations and bars. The latter also provided the highest amounts of energy among the GMP-based products. This finding may be related to a higher risk of developing overweight and obesity. The greater palatability of these GMP-based PSs, combined with improved nutritional quality, could not only improve adherence to diet therapy but also reduce the incidence of obesity-related comorbidities in PKU.

## 1. Introduction

Phenylketonuria (PKU), OMIM: 261600, is an autosomal recessive disorder of phenylalanine (Phe) metabolism characterised by high Phe concentrations in the blood that cause brain dysfunction and other clinical manifestations [1]. Infants affected by PKU, identified through newborn screening, must be immediately treated to avoid the accumulation of dietary Phe in the blood and brain, which can cause cognitive impairments such as severe intellectual disability, epilepsy, behavioural and psychiatric disorders, and psychomotor delays [2]. Good neurocognitive and psychological results can be achieved with a lifelong adherence to a controlled, low-Phe diet, which represents the primary treatment for PKU patients from birth to adulthood. Dietary management of PKU is based on three principles: natural protein restriction, in order to limit Phe intake; consumption of low-protein food products (LPFs); and integration with Phe-free protein substitutes (PSs), in order to guarantee the correct intake of all of the other amino acids contained in natural proteins. According to the European Guidelines for PKU, the total intake of protein consumed should be 40% higher than the FAO/WHO/UNU safe levels of protein intake [1]. Moreover, the exclusion of specific food groups from the diet poses a risk for deficiency in several micronutrients, such as vitamin B12, long-chain polyunsaturated fatty acids (LC-PUFAs), iron, selenium, and zinc. Therefore, supplementation with the lacking vitamins and minerals is needed. This straight diet can be very demanding to follow due to the dullness of dietary routine and the low palatability of PSs, especially for adolescents, for whom a decrease in dietary compliance has been observed [3]. Depending on the severity of PKU, PSs can supply up to 80% of the total daily protein requirement with a well-planned timing and intake, in order to maintain a positive nitrogen balance, portioning the PSs—and, thus, the protein equivalents (P.Eq.) intake—throughout the day. In children with PKU, daily nutrition and, thus, PS composition should meet the requirements for normal growth, ensuring metabolic control, while also providing an adequate tyrosine intake and preventing nutritional deficiency [4].

During the last 20 years, food processing technology has made great progress, widening the formulations of products like powders, ready-to-drink formulations, spoonable products, tablets, or bars, as well as their nutritional composition and the ingredients employed [5], while also improving their palatability. Moreover, among the new PSs available for PKU management, casein glycomacropeptide (CGMP or GMP)-based PSs are gaining in significance. GMP, a natural 64-amino acid glycophosphopeptide derived from kappa-casein and extracted from bovine milk whey, is employed in the treatment of PKU because of its naturally low Phe content [6,7]. In its pure form, GMP is rich in some large neutral amino acids (LNAAs), such as threonine and isoleucine, but does not contain aromatic amino acids such as tryptophan, Phe, tyrosine, or cysteine [8]; however, the final product obtained after its isolation from whey contains residual Phe. As GMP shows inadequate amounts of histidine, leucine, methionine, tryptophan, and tyrosine, GMP-based products must be integrated with free essential amino acids to overcome this lack. Moreover, GMP-based formulations have good palatability, reaching higher satisfaction levels when compared to standard amino acid substitutes, which are notoriously bitter in taste [9,10]. Indeed, the bitter taste and strong odour of standard PSs are known to impact long-term adherence to diet therapy, especially during adolescence [11,12]. Moreover, GMP has an impact on breath malodour, typically associated with PS consumption [13]. The current European Guidelines for PKU do not report a statement concerning the minimum age for the use of GMP-based PSs, but in the actual market these products are indicated for patients aged more than 3 years. In Italy, the availability of GMP-based PSs is growing but is still not uniform [5]. They are usually supplemented with vitamins, minerals, and trace elements, but little research has been carried out in terms of nutritional composition.

This work aims to analyse the differences in terms of energy, macronutrients, micronutrients, and other functional components among the GMP-based PSs in different formulations (ready to drink, powdered, and bars) currently available for the dietary management of children with PKU in Italy. Secondarily, we will assess the quality of these products, comparing their nutritional composition with that of standard amino acid (L-AA) mixtures ordinarily employed for the dietary management of PKU.

## 2. Materials and Methods

From May to September 2023, we asked the companies producing GMP-based PSs available in Italy for PKU to send updated and detailed nutritional composition data of these products. All GMP-based PSs available in Italy were included in this review and then compared in terms of nutritional composition according to their formulation (powdered, ready to drink, and bars) per 100 g or 100 mL of product. GMP-based PSs were also compared per 10 g of protein equivalents (P.Eq.) to assess energy, total fats, saturated fatty acids, carbohydrates, sugars, fibre, and salt contents. We compared the nutritional composition of 10 g of P.Eq. of GMP-based PS to the nutritional composition of 10 g of P.Eq. of the mean nutritional composition of three L-AA PSs used for the management of PKU in children older than 3 years. The three L-AA PSs were selected by a random draw from the https://www.browserling.com/tools/random-number (Accessed on 20 November 2023) site after numbering all of the commercially available L-AA PSs in Italy for children older than 3 years.

## 3. Results

Thirty-five GMP-based PSs produced by six different companies were included in this review. Twenty-one of these products are powder formulations (produced by six companies), eight are ready to drink (produced by two companies), and six are bars (produced by two companies). Tables S1–S3 show the nutritional composition of GMP-based powdered, ready-to-drink, and bar PSs, respectively, divided by producer. Table S4 reports the nutritional composition contained in 100 g of the three selected L-AA powdered PSs indicated for children with PKU from 3 years of age. Table 1, Table 2 and Table 3 show the nutritional comparison for 10 g of P.Eq. for all thirty-five GMP-based PSs, while Table 4 shows the nutritional data for the mean of the three L-AA PSs.

The recommended minimum age for use is 3 years for thirty-three out of thirty-five products, but for six of them (one ready to drink, two bars, and three powdered) caution is recommended in their administration to children aged 3 to 6 years. Only two powdered PSs from the same company (Table 1, Vitaflo International Ltd, Liverpool, UK, GMP-based powders 9 and 10) are recommended for use in children aged 4 years and older.

The analysed products vary greatly in their energy content, with values between 298 kcal/100 g and 400 kcal/100 g for powdered products, between 14 kcal/100 mL and 82 kcal/100 mL for ready-to-drink PSs, and between 313 kcal/100 g and 407 kcal/100 g for bar products. The average energy value of the L-AA PSs is 329 kcal per 100 g. Considering the nutritional values related to 10 g of P.Eq. (Table 1, Table 2, Table 3 and Table 4), the average energy content of the L-AA PSs is 55 kcal; GMP-based bars have from three to more than four times this content, ready-to-drink PSs have from less than twice to three times as much, and the powders’ energy content is variable, being similar to that of L-AA PSs in some products but reaching three times that of L-AA PSs in others.

Among the powdered PSs, the protein equivalents (P.Eq.) content varies between 25 and 67 g/100 g, with a Phe content ranging from 0.8 to 2.1 mg/g of proteins (0.3–1 mg/g of product). The P.Eq. content in the ready-to-drink PSs varies between 2 and 6 g/100 mL, with a Phe content between 1 and 1.8 mg/g of proteins (between 3 and 10.8 mg/100 mL of product). The bars’ P.Eq. content varies between 16.67 and 20 g/100 g, with a Phe content between 1.25 and 2.4 mg/g of proteins (0.2–0.45 mg/g of product). L-AA PSs are characterised by an average P.Eq. content of 59.8 g/100 g.

Considering carbohydrates in powdered products, the content varies from 2.4 g/100 g to 68 g/100 g, while the range of sugars varies from 0.6 g/100 g to 65 g/100 g, and in percentage terms between 6.15% and 95.6% of total carbohydrates. For the ready-to-drink PSs, the carbohydrate content varies from 1.4 g/100 mL to 10 g/100 mL, while the range of sugars varies from 0 g/100 mL (Table S1, Ajinomoto Cambrooke, Inc. Ayer, MA 01432 USA, GMP-based ready-to-drink products 6 and 7) to 8 g/100 mL, and in percentage terms between 0% and 88.2% of total carbohydrates. The carbohydrate content of the GMP-based bars varies from 47 g/100 g to 56 g/100 g, while the range of sugars varies in absolute terms from 12.4 g/100 g to 35 g/100 g, and in percentage terms between 25% and 73% of total carbohydrates. Four out of six bars declare the presence of polyols in quantities varying between 1 and 9 g/100 g of product. The amount of fibre varies between 0 and 14 g/100 g for powdered products, but for four out of twenty-one (19%) powdered PSs the fibre content is not expressed on the technical data sheet. The powdered PSs with the highest fibre contents per 10 g of P.Eq. (powdered products 4, 5, and 6) contain only soluble fibre. The ready-to-drink PSs’ fibre content ranges from 0 to 0.9 g/100 mL, while it varies between 4/100 g and 8.4 g/100 g for bars. In comparison to the analysed GMP products, the average contents of carbohydrates, sugars, and fibre in L-AA PSs are 17.53 g/100 g, 4 g/100 g, and 7.61 g/100 g, respectively, albeit with great variability among the three L-AA PSs considered. As far as the nutritional values related to 10 g of P.Eq. are concerned, the average carbohydrate content of the L-AA PSs is 2.93 g; the GMP-based bars have more than 10 times this content, the ready-to-drink formulations have from double to almost 8 times as much, and the powders have from 8 times less to 10 times more than this amount. For sugars, the L-AA PSs have an average content of 0.67 in 10 g of P.Eq.; the sugar content is almost 30 times as high in bars and more than 25 times as high in some ready-to-drink PSs, whereas the powdered PSs show a more heterogeneous content, varying from one-quarter of that of L-AA PSs up to 40 times more (Table S1, Cambrooke, GMP-based powdered products 20 and 21). Lastly, the average fibre content in the L-AA PSs is 1.27 g in 10 g of P.Eq.; while the ready-to-drink PSs have similar contents, only two of the bars have four times this content (Table S3, Medifood, Piam Farmaceutici S.P.A., 16121 Genova, Italy, GMP-based bars 1 and 2). For the powdered PSs, the fibre content is highly variable.

Looking at lipids in the powdered products, the total fat contents vary from 0 g/100 g to 11.7 g/100 g, while the saturated fat range varies in absolute terms from 0/100 g to 4.6 g/100 g, with a saturated/total ratio between 0 and 0.5. For the ready-to-drink PSs, the total fat content varies from 0 g/100 mL to 2 g/100 mL, while the saturated fat range varies from 0 g/100 mL to 0.8 g/100 mL, with a saturated/total ratio between 0 and 0.4. For the bars, the total fat content varies from 1.8 g/100 g to 14.8 g/100 g, while the content of saturated fat ranges from 0.8 g/100 g to 13 g/100 g, with a saturated/total ratio between 0.44 and 0.87. Only fourteen out of the thirty-five GMP-based products (thirteen powders and one liquid) describe the exact contents of monounsaturated and polyunsaturated fatty acids. Among all of the PSs, docosahexaenoic acid (DHA) is contained in only seventeen out of thirty-five (48.57%, of which fourteen powdered and three ready to drink), with values between 62 and 314 mg/100 g for powdered PSs, and from 24 to 44 mg/100 mL for ready-to-drink PSs. Bars do not contain DHA. The L-AA PSs have an average content of less than 1.5 g/100 g for lipids and less than 0.52 g/100 g for saturated fats; DHA (540 mg/100 g) is present only in one PS. The average contents of total fats and saturated fatty acids per 10 g of P.Eq. in the L-AA PSs are 0.25 g and 0.09 g, respectively; the GMP-based bars can have up to 30 times more lipids and up to almost 80 times more saturated fatty acids; in ready-to-drink PSs, the contents are more heterogeneous, varying from products without lipids to products with 15 times the contents of total fats and saturated fatty acids in the L-AA PSs. Some of the GMP-based powdered have almost no lipids, while others have more than 10 times the contents of total fats and saturated fatty acids in the L-AA PSs.

The salt content varies between 0.82 and 2.2 g/100 g for the powdered products, from 0.16 to 0.63 g/100 mL for the ready-to-drink PSs, and from 0.35 to 0.70 g/100 g for the bars. The average salt content in the L-AA PSs is 0.24 g/100 g. Per 10 g of P.Eq., the average salt content is 0.04 g; the bars have up to 8 times this salt content, while one ready-to-drink PS and two of the powders have more than 20 times the salt content.

Only five powdered GMP-based PSs from Company F contain Bacillus Coagulans, a microorganism with probiotic activity, while none of the other GMP-based or L-AA PSs contain modulators of the gut microbiota.

## 4. Discussion

The analysis of the GMP-based products included in this review revealed a great heterogeneity not only among the different formulations (powdered, ready to drink, and bars), but also within the same formulation groups, in terms of energy and macronutrient composition. Sugars and saturated fatty acids showed not only the most variable values among GMP-based products, but also the highest values compared to the L-AA PSs. The sugar contents appeared to be heterogeneous but high in both ready-to-drink and bar PSs. GMP-based bars, in addition to having a non-negligible energy intake, contained more sugars and saturated fatty acids for the same quantity of product, along with higher P.Eq. contents compared to the powdered or liquid formulations. The bars were also rich in numerous ingredients compared to other GMP-based products. Some of these ingredients, even if present in traces, may represent a risk and source of insecurity for patients with food allergies. In fact, several of these ingredients are recognised as allergens, representing a risk for children with PKU. The higher energy and sugar contents in GMP-based PSs compared to L-AA PSs per 10 g of P.Eq., especially for bars, associated with the absence or low content of fibre in most PSs, may be related to the energy and sugar intake exceeding nutritional requirements, thus increasing the risk of developing overweight and obesity or non-communicable diseases (NCDs) in adulthood.

Moreover, the salt content of the GMP-based PSs was higher than that of the L-AA PSs, especially for ready-to-drink PSs. Elevated salt and, thus, sodium intake represents a known risk factor for hypertension, as well as cardiovascular and kidney diseases. Regulation (EC) No 1924/2006 of the European Parliament and of the Council of 20 December 2006 on nutrition and health claims made on foods defines a food as low in sodium/salt if it contains no more than 0.12 g of sodium (0.3 g of salt) per 100 g/100 mL, and very low in sodium/salt if it contains no more than 0.04 g of sodium (0.1 g of salt) per 100 g/100 mL. In our review, most of the powdered PSs exceeded 0.3 g of salt per 100 g.

These nutritional analyses do not take into account the different recommendations for mixing GMP-based PSs with different foods. While bars and ready-to-drink PSs can be consumed immediately by PKU patients, powdered products need to be mixed in other drinks (e.g., water, fruit juice, flavoured drinks) or semi-solids (e.g., cream, sauce), or as ingredients in solid food (e.g., pancakes, toast), according to the directions for preparation and usage provided by each company. This could lead not only to a modified taste, but also to different nutritional intakes. Moreover, GMP-based PSs are usually consumed by PKU patients together with other PSs, such as L-AA PSs. For this reason, it is essential that a dietician carefully assesses the patient’s diet plan, monitoring intakes over time, to ensure the respect of recommended daily allowances (RDAs) or dietary reference values (DRVs) for age and sex. Table 5 reports the DRVs of the European Food Safety Authority (EFSA) for healthy children. In PKU, protein requirements are increased compared to healthy children, and PSs can supply from 52 to 80% of the total protein requirements [1], depending on the portion consumed. Moreover, PSs can also partly meet requirements for energy, carbohydrates, and lipids, depending on the specific composition of the product. As most of these products are supplemented with minerals and vitamins, they also may contribute to the prevention of micronutrient deficiencies; therefore, the addition of other supplements must be specifically evaluated according to the nutritional composition of the PS(s) consumed.

### 4.1. Risk of Overweight and Obesity in PKU

The carbohydrate and fat contents in the GMP-based PSs analysed here are generally higher than those reported in free L-AAs, leading to a higher daily energy intake in children with PKU who consume GMP-based PSs alone or in combination with L-AAs, in comparison with patients only consuming L-AAs as PSs. The higher carbohydrate and energy contents potentially lead to a higher risk of obesity. Although no scientific evidence indicates that a Phe-restricted diet represents a risk factor for developing overweight itself, a recent systematic review showed that patients with classical PKU adherent to diet, especially females, had a significantly higher BMI than healthy controls, probably due to a higher calorie content in these patients’ diets in order to prevent catabolism, which causes higher blood Phe levels [14]. GMP-based PSs are designed for children with PKU over 3 years of age. Nutrients and Energy for Italian Population (LARN) indicates a reference intake range for lipids of 20–35% of the total energy requirement for children older than 3 years, while in infants and children under 3 years of age the recommended intake of lipids is higher. Children with PKU should follow the same nutritional guidelines for lipids as healthy children, promoting a normal-lipid diet. GMP-based PSs, and especially bars, are not fully adequate to meet the recommended age reference intakes, leading to a higher fat intake. In addition, almost all nutrition labels do not show the lipidogram; therefore, it is not possible to assess the quality of fatty acids.

### 4.2. Impact on Carbohydrate Metabolism and Glycaemia

Previous studies have suggested an association between PKU dietary treatment and higher glycaemic index (GI) and triglyceride glucose index (TyG), which seem to mainly reflect peripheral insulin resistance [15]. These findings have been attributed to the higher GI carbohydrate intake in PKU children than in healthy peers [16]. An observational case–control study in children with PKU found that dietary GI and glycaemic load (GL) are higher in children with PKU than in healthy children [16]. Moreover, focusing on fibre intake, even though children with PKU consume more vegetables and fibre, especially soluble fibre, their gut microbiota composition is not favourable, with important alterations in Firmicutes phylum and a reduction in butyrate-producing genera [17,18]. A recent systematic review and meta-analysis examining PKU microbiota [19] highlighted the impact of diet in PKU, together with the administration of PSs and/or other supplementations, on the alterations in microbial communities, potentially influencing not only carbohydrate metabolism but also AA biosynthesis and gastroenterological symptomatology. At present, no data are available on the GI and GL of PSs for PKU, but the intake of Phe-free amino acids has been shown to cause a significantly higher insulin peak after the intake of Phe-free L-AA PSs in both humans and rats [20]. A putative explanation is that amino acids, especially leucine, which is contained in large amounts in Phe-free amino acid supplements, act as important triggers of insulin secretion [21]. In a study on a murine model, Pena et al. [22] analysed the chronic effects of casein (CAS), free amino acids (L-AAs), and GMP on glycaemic metabolism by assessing its biochemical markers in blood. No significant differences in glycaemia were observed among the three treatments, while insulin levels were significantly higher in rats fed with GMP than those fed with L-AAs, in contrast to what was expected. This finding is probably because the post-absorptive levels of amino acids were lower, and the insulin levels were only measured at the end and not during the experiment. In fact, the GMP diet contained similar amounts of leucine to those from L-AAs, with the exception that around half of the amount came from intact proteins and the other half from free amino acids. Furthermore, isoleucine and valine, which are insulinogenic amino acids as well, only came from original GMP without additional amino acids. However, regarding HOMA-IR, a surrogate measure of insulin sensitivity, a significant difference was observed between GMP and L-AAs, indicating that rats fed with L-AAs were 3-fold more insulin-resistant than those fed with GMP. In fact, those products from which fats are removed often have more added sugars. Future research should consider the insulinaemic index and not just the glycaemic load of PSs. Always focusing on the impact of GMP-based PSs on glucose metabolism, it must be considered that GMP is a casein derived from whey. A double-blinded randomised trial confirmed that whey protein stimulates fasting insulin while casein stimulates circulating insulin-like growth factor 1 (IGF-1), highlighting that milk protein fractions can have different effects [23].

### 4.3. GMP-Based PSs and Blood Phe Levels

Among the PSs analysed, the Phe content ranged from 0.03 mg/g (ready-to-drink PSs) to 1 mg/g (powdered PSs) of product, with lower concentrations in ready-to-drink formulations and higher concentrations in bars. Although GMP has a residual Phe content and, thus, GMP-based PSs are not Phe-free, studies regarding the effects of GMP on blood Phe levels have shown no significant increase in comparison with L-AAs [9,11,12,24]. However, two studies from the same centre [25,26] demonstrated a statistically significant increase in Phe levels in children, suggesting the importance of close monitoring of Phe blood values when GMP is administered to paediatric populations with classical PKU. Conversely, GMP’s residual Phe showed a lower impact on Phe blood levels in patients receiving a combined pharmacological treatment, or in adolescents, who should maintain Phe values within a less straight range. These values are in line with a study performed by Van Calcar et al. [27], according to which a residual Phe in GMP of 4 mg/g of product was too high; therefore, they refined the GMP composition in order to administer a maximum of 2 mg/g of product. It has been postulated that GMP is able to reduce amino acid absorption, unlike L-AAs, which are quickly absorbed by the small intestine, with a rapid and short-lasting peak in the blood [28]. Van Calcar et al. [27] showed minor variability in blood Phe after GMP consumption in comparison to L-AA consumption in 11 subjects aged 11–31 years after an overnight fast, supporting a slower release of amino acids in GMP users. Furthermore, a more sustained release of amino acids may improve nitrogen balance with reduced urea production, contributing to better growth [27,29,30,31]. Considering Phe tolerance in PKU, it is important to state that lifetime natural protein (NP) tolerance has been little described, regardless of the fact that excessive restriction contributes to poor growth and nutritional status [32]. A retrospective longitudinal study examining NP tolerance showed that the 65% of PKU patients older than 12 years could tolerate more NP than the quantity prescribed [33]. In light of this, GMP-based PSs could play an important role in promoting dietary adherence, especially in adolescents.

### 4.4. Impact of GMP on Gut Microbiota Composition

The content of sugars, which is highly variable among the presented PSs, should also be taken into consideration for its potential modulation of the microbiota. Indeed, results from studies conducted on both murine PKU models [34] and PKU patients [35] suggest a probiotic role of GMP. This effect may be due to its structure, characterised by extensive glycosylation with sugars (sialic acid, galactosyl, and *N*-acetylgalactosamine), which are substrates for some specific beneficial bacteria such as Lactobacillus and Bifidobacteria. In a pilot study, the effect of GMP intake on the gut microbiota was studied in four adult and five paediatric PKU patients. A specific prebiotic effect on butyrate-producing taxa (*Agathobacter* spp. and, to a lesser extent, Subdoligranulum) was observed, without dramatic changes in the commensal microbiota [35]. In our review, only one company had added a probiotic bacterium to some of its products; this spore-forming bacterial species, once active in the small intestine after germination, has been shown to aid in the digestion of carbohydrates and proteins by improving their absorption and utilisation [36]. Studies on Bacillus Coagulans’s impact on the gut microbiota at paediatric age have also been published [37]. Furthermore, an immunomodulatory influence was observed in murine models, documented by reduced plasmatic concentrations of IFN-γ, TNF-α, IL-1β, and IL-2 [34]. The best impact on the composition of the gut microbiota is provided by products with both soluble and insoluble fibre; the nature of the fibre should be better specified on the nutritional labels of products in order to guarantee a beneficial effect [38].

### 4.5. Advantages and Beneficial Effects of GMP-Based PSs

One of the most evident advantages of using GMP-based PSs is their greater palatability in comparison with L-AAs. Indeed, L-AAs are well known to have a bitter flavour, with an aftertaste that is poorly tolerated by both children and adults [13]. Several studies have demonstrated an improved taste profile in GMP-based products, which would favour better compliance to a lifelong diet [9,11,12,24,25,39,40], although no significant differences have been found in the contents of volatile organic compounds in the fasting and postprandial exhaled breath samples of children with PKU taking GMP or L-AAs [13].

According to evidence from non-PKU conditions or PKU animal models, the partial replacement of L-AAs with GMP as a PS in the diets of children with PKU seems to bring several clinical benefits, thanks to its composition and bioactive properties [4]. GMP may play an anti-inflammatory and antioxidative role against the intestinal hydrogen peroxide (H_2_O_2_)- and lipopolysaccharide (LPS)-induced oxidative stress, worsened by free L-AAs, potentially restoring a physiological condition in the gut, as recently demonstrated in vitro [41]. Through this mechanism, GMP may conversely represent a protective factor against NCDs; therefore, multidisciplinary lifelong dietary management may be helpful to set a correct balance between the intakes of GMP and free L-AAs.

Another functional property of PSs in PKU is providing patients with large neutral amino acids (LNAAs). The LNAA transporter (LAT1) is responsible for the conveyance of Phe into the brain. LNAAs compete with Phe for LAT1, preventing excessive Phe from entering the brain [42]. GMP is naturally rich in threonine and isoleucine, and GMP products for PKU patients are usually integrated with tryptophan and tyrosine. All of the GMP products that we have reported in this review contain threonine, isoleucine, tryptophan, and tyrosine, with variable concentrations among the same categories. These LNAAs may play an important role in preventing neurocognitive damage through this mechanism [42].

Moreover, some evidence suggests that children using GMP as their only source of protein substitute are taller, with greater lean body mass and lower fat mass [4]. Non-significant differences in terms of bone mass, density, and geometry have been demonstrated between children with PKU consuming L-AAs or GMP [43], although an increase in the serum bone marker procollagen type 1 *N*-terminal propeptide (P1NP) was observed in children taking only GMP as a protein substitute, in comparison with children taking only L-AAs. A recent pilot study reported an amelioration of calcium phosphate homeostasis with an increase in plasmatic vitamin D and a decrease in alkaline phosphatase in nine PKU patients, including five children, after a 6-month GMP intake [35].

Finally, recent studies have shown that GMP is able to ameliorate the postprandial glucose homeostasis in obese postmenopausal women, together with promoting satiety and increasing the concentration of amylin, a neuroendocrine satiety hormone. Moreover, beneficial alterations in gut microbiota composition after GMP intake in women with obesity have also been described [44,45]. Future studies should explore the anti-obesity effects and metabolic comorbidities associated with GMP-based products in children with metabolically unhealthy obesity (MUO), but more attention to their nutritional composition—in terms of sugars, fibre, and total fats—is needed, together with the identification of the best formulation.

**Figure 1:**
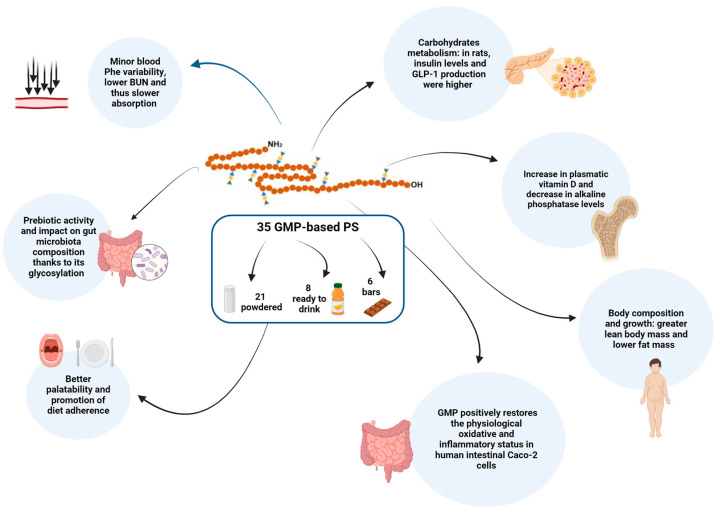
Graphical representation of the scientifically proven health outcomes of GMP.

Figure 1 summarizes the multiple effects of GMP in terms of scientifically proven health outcomes.

## 5. Conclusions

Our analysis shows heterogeneity in terms of nutritional composition among GMP-based PSs, both within the same formulations (powdered, ready to drink, and bars) and between different formulations. Despite the potential benefits of GMP-based PSs, which have already been documented in several clinical studies, the high energy density and low nutritional quality of some of these products could lead to the nutritional recommendations being exceeded, causing an imbalance between the benefits and merits of these products. Given the well-known negative impact of free amino acid PSs on protein status and their poor acceptability, it is necessary for industries to focus on improving the nutritional composition of GMP products for children with PKU, lowering sugar and fat contents and increasing the fibre content, preferably choosing as many natural ingredients as possible. GMP-based PSs, being more palatable, can improve adherence to diet therapy but need to be improved in terms of nutritional composition and quality in order to reduce the incidence of NCDs in PKU.

## Figures and Tables

**Table 1:** Food components contained in 10 g of protein equivalents (P.Eq.) for GMP-based protein substitutes—powdered. If nutritional data were not present on the nutritional label, the term “Not Declared” (ND) was used.

Content per 10 g of P.Eq.	Unit	MEDIFOOD		NUTRICIA	MAMOXI			MEVALIA				VITAFLO	
		Powdered 1	Powdered 2	Powdered 3	Powdered 4	Powdered 5	Powdered 6	Powdered 7		Powdered 8		Powdered 9	Powdered 10
Energy	Kcal	79.50	79.00	128.00	54.18	54.18	54.18	74.19		78.05		60.36	60.36
Total fats	g	0.05	0.05	3.90	0.36	0.36	0.36	0.40		0.37		0.84	0.84
Saturated fatty acids	g	0.03	0.03	0.93	0.18	0.18	0.18	0.16		0.10		0.18	0.18
Carbohydrates	g	9.78	9.70	12.50	1.45	1.45	1.45	7.44		8.05		3.21	3.21
Sugars	g	0.60	0.60	8.67	0.15	0.15	0.15	4.42		5.37		1.12 (1.42)	1.125
Fibre	g	ND	ND	1.50	2.55	2.55	2.55	0.70		0.73		ND	ND
Salt	g	0.21	0.21	0.43	0.27	0.27	0.27	0.19		0.23		0.36	0.36
**Content per 10 g of P.Eq.**	**Unit**	**CAMBROOKE**											
		**Powdered 11**	**Powdered 12**	**Powdered 13**	**Powdered 14**	**Powdered 15**	**Powdered 16**	**Powdered 17**	**Powdered 18**		**Powdered 19**	**Powdered 20**	**Powdered 21**
Energy	Kcal	105.26	101.32	105.26	50.00	50.00	54.60	55.47	52.03		55.16	146.00	146.00
Total fats	g	2.89	3.03	2.89	0.63	0.63	1.00	1.02	0.95		1.00	0.00	0.00
Saturated fatty acids	g	1.21	1.18	1.21	0.10	0.10	0.13	0.11	0.09		0.11	0.00	0.00
Carbohydrates	g	9.74	13.16	13.16	0.36	0.36	0.83	0.86	0.86		0.84	27.20	27.20
Sugars	g	3.16	3.42	3.42	0.12	0.12	0.10	0.13	0.13		0.09	26.00	26.00
Fibre	g	0.00	0.03	0.00	0.00	0.00	0.16	0.16	0.16		0.16	0.00	0.00
Salt	g	0.47	0.32	0.32	0.33	0.33	0.33	0.34	0.34		0.34	0.80	0.80

Explanation of GMP-based protein substitutes—powdered. MEDIFOOD. GMP-based powdered 1: Afenil GMP Up Shake milk; GMP-based powdered 2: Afenil GMP Up Shake orange. NUTRICIA. GMP-based powdered 3: PKU GMPro. MAMOXI. GMP-based powdered 4: XPhe Enjoy GMP Fibre; GMP-based powdered 5: XPhe Enjoy GMP Chocolate Fibre; GMP-based powdered 6: XPhe Enjoy GMP Vanilla Fibre. MEVALIA. GMP-based powdered 7: PKU GMP Power; GMP-based powdered 8: PKU GMP Power Pina Colada. VITAFLO. GMP-based powdered 9: PKU Sphere 15; $ PKU Sphere 15 Chocolate. Recommended serving of 27 g. GMP-based powdered 10: PKU Sphere 20. CAMBROOKE. GMP-based powdered 11: Glytactin Bettermilk 15; GMP-based powdered 12: Glytactin Bettermilk 15 Orange; GMP-based powdered 13: Glytactin Bettermilk 15 Strawberry; GMP-based powdered 14: Glytactin Build 10; GMP-based powdered 15: Glytactin Build 20;. GMP-based powdered 16: Glytactin Build 20 Chocolate; GMP-based powdered 17: Glytactin Build 20 Raspberry Lemonade; GMP-based powdered 18: Glytactin Build 20 Vanilla; GMP-based powdered 19: Glytactin Build 20 Smooth; GMP-based powdered 20: Glytactin Restore Powder Orange; GMP-based powdered 21: Glytactin Restore Powder Berry.

**Table 2:** Food components contained in 10 g of protein equivalents (P.Eq.) for GMP-based protein substitutes—ready to drink.

Content per 10 g of P.Eq.	Unit	NUTRICIA	CAMBROOKE						
		Ready to Drink 1	Ready to Drink 2	Ready to Drink 3	Ready to Drink 4	Ready to Drink 5	Ready to Drink 6	Ready to Drink 7	Ready to Drink 8
Energy	Kcal	112.50	161.50	161.50	136.67	136.67	80.00	80.00	70.00
Total fats	g	4.00	3.50	3.50	3.33	3.33	2.33	2.33	0.00
Saturated fatty acids	g	0.40	1.00	1.00	1.33	1.33	0.33	0.33	0.00
Carbohydrates	g	8.50	22.50	22.50	16.67	16.67	4.83	5.00	7.00
Sugars	g	7.50	17.50	17.50	13.33	13.33	0.00	0.00	5.00
Fibre	g	0.93	1.00	1.00	0.67	0.67	1.50	1.50	0.00
Salt	g	0.40	0.63	0.63	0.40	0.40	0.50	0.50	0.95

Explanation of GMP-based protein substitutes—ready to drink. NUTRICIA. GMP-based ready to drink 1: PKU GMPro LQ. CAMBROOKE. GMP-based ready to drink 2: Glytactin RTD 10 Smooth; GMP-based ready to drink 3: Glytactin RTD 10 Chocolate; GMP-based ready to drink 4: Glytactin RTD 15 Smooth; GMP-based ready to drink 5: Glytactin RTD 15 Chocolate; GMP-based ready to drink 6: Glytactin RTD 15 Lite Mocha; GMP-based ready to drink 7: Glytactin RTD 15 Lite Vanilla; GMP-based ready to drink 8: Glytactin Restore Lite 10 Tangerine.

**Table 3:** Food components contained in 10 g of protein equivalents (P.Eq.) for GMP-based protein substitutes—bars.

Content per 10 g of P.Eq.	Unit	MEDIFOOD		CAMBROOKE			
		Bar 1	Bar 2	Bar 3	Bar 4	Bar 5	Bar 6
Energy	Kcal	156.00	210.00	210.00	213.33	220.00	220.00
Total fats	g	0.90	4.30	6.00	5.33	8.00	8.00
Saturated fatty acids	g	0.40	3.40	4.00	4.00	7.00	6.00
Carbohydrates	g	24.70	30.20	30.00	30.00	26.00	22.67
Sugars	g	6.20	12.00	14.00	12.67	19.00	16.67
Fibre	g	5.00	5.00	2.00	2.00	2.00	2.00
Salt	g	0.21	0.21	0.30	0.26	0.30	0.33

Explanation of GMP-based protein substitutes—bar. MEDIFOOD. GMP-based bar 1: AFENIL GMP UP BAR CREAM-MOU; GMP-based bar 2: AFENIL GMP UP BAR COCONUT. CAMBROOKE. GMP-based bar 3: Glytactin Complete Cocoa 10; GMP-based bar 4: Glytactin Complete Cocoa 15; GMP-based bar 5: Glytactin Complete Fruit 10; GMP-based bar 6: Glytactin Complete Fruit 15.

**Table 4:** Food components contained in the mean of 10 g of protein equivalents (P.Eq) for three different L-AA powdered protein substitutes available for children with PKU from 3 years of age.

Content per 10 g of P.Eq.	Unit	Mean of 3 L-AA PSs
Energy	Kcal	55.07
Total fats	g	0.25
Saturated fatty acids	g	0.09
Carbohydrates	g	2.93
Sugars	g	0.67
Fibre	g	1.27
Salt	g	0.04

Explanation of L-AA protein substitutes—powdered. L-AA protein substitutes: MEDIFOOD AFENIL BUDDY, MAMOXI XPHE SMART K, and NUTRICIA PKU LOPHLEX NEUTRAL.

**Table 5:** European Food Safety Authority (EFSA) dietary reference values (DRVs) for children and adolescents for total fats, saturated fatty acids, carbohydrates, fibre, and sodium.

EFSA—DRV	Male and Female
Total fats	Reference intake (RI) 2–3 years 35–40 of energy intake 4–17 years 20–35 of energy intake
Saturated fatty acids	Adequate intake (AI) All ages: as low as possible (ALAP)
Carbohydrates	Reference intake (RI) 1–17 years 45–60% of energy intake
Fibre	Adequate intake (AI) 1–3 years 10 g/day 4–6 years 14 g/day 7–10 years 16 g/day 11–14 years 19 g/day 15–17 years 21 g/day
Sodium	Safe and adequate intake 1–3 years 1.1 g/day 4–6 years 1.3 g/day 7–10 years 1.7 g/day 11–17 years 2 g/day

## Supplementary Materials

The following supporting information can be downloaded at: https://www.mdpi.com/article/10.3390/nu16070956/s1, Table S1: Food components contained in 100 g of GMP-based protein substitutes—powdered. If nutritional data were not present on the nutritional label, the term “Not Declared” (ND) was used. Table S2: Food components contained in 100 mL of GMP-based protein substitutes—ready to drink. If nutritional data were not present on the nutritional label, the term “Not Declared” (ND) was used. Table S3: Food components contained in 100 g and per portion of GMP-based protein substitutes—bars. If nutritional data were not present on the nutritional label, the term “Not Declared” (ND) was used. Table S4: Mean of the food components contained in 100 g of 3 different L-AA powdered protein substitutes available for children with PKU from 3 years of age. If nutritional data were not present on the nutritional label, the term “Not Declared” (ND) was used.

## References

[1] van Wegberg A.M.J., MacDonald A., Ahring K., Bélanger-Quintana A., Blau N., Bosch A.M., Burlina A., Campistol J., Feillet F., Giżewska M. (2017). The Complete European Guidelines on Phenylketonuria: Diagnosis and Treatment. Orphanet J. Rare Dis..

[2] van Spronsen F.J., Blau N., Harding C., Burlina A., Longo N., Bosch A.M. (2021). Phenylketonuria. Nat. Rev. Dis. Primers.

[3] MacDonald A., van Wegberg A.M.J., Ahring K., Beblo S., Bélanger-Quintana A., Burlina A., Campistol J., Coşkun T., Feillet F., Giżewska M. (2020). PKU Dietary Handbook to Accompany PKU Guidelines. Orphanet J. Rare Dis..

[4] Daly A., Ilgaz F., Pinto A., MacDonald A. (2024). Casein glycomacropeptide in phenylketonuria: Does it bring clinical benefit?. Curr. Opin. Clin. Nutr. Metab. Care.

[5] Pena M.J., de Almeida M.F., van Dam E., Ahring K., Bélanger-Quintana A., Dokoupil K., Gokmen-Ozel H., Lammardo A.M., MacDonald A., Robert M. (2016). Protein substitutes for phenylketonuria in Europe: Access and nutritional composition. Eur. J. Clin. Nutr..

[6] Lim K., van Calcar S.C., Nelson K.L., Gleason S.T., Ney D.M. (2007). Acceptable Low-Phenylalanine Foods and Beverages Can Be Made with Glycomacropeptide from Cheese Whey for Individuals with PKU. Mol. Genet. Metab..

[7] Nakano T., Ozimek L. (2014). A sialic acid assay in isolation and purification of bovine k-casein glycomacropeptide: A review. Recent. Pat. Food Nutr. Agric..

[8] Neelima, Sharma R., Rajput Y.S., Mann B. (2013). Chemical and Functional Properties of Glycomacropeptide (GMP) and Its Role in the Detection of Cheese Whey Adulteration in Milk: A Review. Dairy Sci. Technol..

[9] Ney D.M., Stroup B.M., Clayton M.K., Murali S.G., Rice G.M., Rohr F., Levy H.L. (2016). Glycomacropeptide for Nutritional Management of Phenylketonuria: A Randomized, Controlled, Crossover Trial. Am. J. Clin. Nutr..

[10] Giovannini M., Verduci E., Salvatici E., Paci S., Riva E. (2012). Phenylketonuria: Nutritional Advances and Challenges. Nutr. Metab..

[11] Zaki O.K., El-Wakeel L., Ebeid Y., Ez Elarab H.S., Moustafa A., Abdulazim N., Karara H., Elghawaby A. (2016). The Use of Glycomacropeptide in Dietary Management of Phenylketonuria. J. Nutr. Metab..

[12] Pena M.J., Pinto A., Daly A., MacDonald A., Azevedo L., Rocha J.C., Borges N. (2018). The Use of Glycomacropeptide in Patients with Phenylketonuria: A Systematic Review and Meta-Analysis. Nutrients.

[13] Tiele A., Daly A., Hattersley J., Pinto A., Evans S., Ashmore C., MacDonald A., Covington J.A. (2019). Investigation of Paediatric PKU Breath Malodour, Comparing Glycomacropeptide with Phenylalanine Free L-Amino Acid Supplements. J. Breath Res..

[14] Rodrigues C., Pinto A., Faria A., Teixeira D., van Wegberg A.M.J., Ahring K., Feillet F., Calhau C., MacDonald A., Moreira-Rosário A. (2021). Is the Phenylalanine-Restricted Diet a Risk Factor for Overweight or Obesity in Patients with Phenylketonuria (PKU)? A Systematic Review and Meta-Analysis. Nutrients.

[15] Irace C., Carallo C., Scavelli F.B., De Franceschi M.S., Esposito T., Tripolino C., Gnasso A. (2013). Markers of Insulin Resistance and Carotid Atherosclerosis. A Comparison of the Homeostasis Model Assessment and Triglyceride Glucose Index. Int. J. Clin. Pract..

[16] Moretti F., Pellegrini N., Salvatici E., Rovelli V., Banderali G., Radaelli G., Scazzina F., Giovannini M., Verduci E. (2017). Dietary Glycemic Index, Glycemic Load and Metabolic Profile in Children with Phenylketonuria. Nutr. Metab. Cardiovasc. Dis..

[17] Bassanini G., Ceccarani C., Borgo F., Severgnini M., Rovelli V., Morace G., Verduci E., Borghi E. (2019). Phenylketonuria Diet Promotes Shifts in Firmicutes Populations. Front. Cell Infect. Microbiol..

[18] Verduci E., Moretti F., Bassanini G., Banderali G., Rovelli V., Casiraghi M.C., Morace G., Borgo F., Borghi E. (2018). Phenylketonuric Diet Negatively Impacts on Butyrate Production. Nutr. Metab. Cardiovasc. Dis..

[19] Ubaldi F., Frangella C., Volpini V., Fortugno P., Valeriani F., Romano Spica V. (2023). Systematic Review and Meta-Analysis of Dietary Interventions and Microbiome in Phenylketonuria. Int. J. Mol. Sci..

[20] Weigel C., Rauh M., Kiener C., Rascher W., Knerr I. (2007). Effects of Various Dietary Amino Acid Preparations for Phenylketonuric Patients on the Metabolic Profiles along with Postprandial Insulin and Ghrelin Responses. Ann. Nutr. Metab..

[21] Pena M.J., Rocha J.C., Borges N. (2015). Amino Acids, Glucose Metabolism and Clinical Relevance for Phenylketonuria Management. Ann. Nutr. Disord. Ther..

[22] Pena M.J., Costa R., Rodrigues I., Martins S., Guimarães J.T., Faria A., Calhau C., Rocha J.C., Borges N. (2021). Unveiling the Metabolic Effects of Glycomacropeptide. Int. J. Mol. Sci..

[23] Hoppe C., Mølgaard C., Dalum C., Vaag A., Michaelsen K.F. (2009). Differential Effects of Casein versus Whey on Fasting Plasma Levels of Insulin, IGF-1 and IGF-1/IGFBP-3: Results from a Randomized 7-Day Supplementation Study in Prepubertal Boys. Eur. J. Clin. Nutr..

[24] Ahring K.K., Lund A.M., Jensen E., Jensen T.G., Brøndum-Nielsen K., Pedersen M., Bardow A., Holst J.J., Rehfeld J.F., Møller L.B. (2018). Comparison of Glycomacropeptide with Phenylalanine Free-Synthetic Amino Acids in Test Meals to PKU Patients: No Significant Differences in Biomarkers, Including Plasma Phe Levels. J. Nutr. Metab..

[25] Daly A., Evans S., Chahal S., Santra S., MacDonald A. (2017). Glycomacropeptide in Children with Phenylketonuria: Does Its Phenylalanine Content Affect Blood Phenylalanine Control?. J. Hum. Nutr. Diet..

[26] Daly A., Evans S., Chahal S., Santra S., Pinto A., Gingell C., Rocha J.C., van Spronsen F., Jackson R., MacDonald A. (2019). The Effect of Glycomacropeptide versus Amino Acids on Phenylalanine and Tyrosine Variability over 24 Hours in Children with PKU: A Randomized Controlled Trial. Nutrients.

[27] van Calcar S.C., MacLeod E.L., Gleason S.T., Etzel M.R., Clayton M.K., Wolff J.A., Ney D.M. (2009). Improved Nutritional Management of Phenylketonuria by Using a Diet Containing Glycomacropeptide Compared with Amino Acids. Am. J. Clin. Nutr..

[28] Wu G. (2009). Amino Acids: Metabolism, Functions, and Nutrition. Amino Acids.

[29] Dangin M., Boirie Y., Garcia-Rodenas C., Gachon P., Fauquant J., Callier P., Ballèvre O., Beaufrère B. (2001). The Digestion Rate of Protein Is an Independent Regulating Factor of Postprandial Protein Retention. Am. J. Physiol. Endocrinol. Metab..

[30] Young V.R., El-Khoury A.E., Raguso C.A., Forslund A.H., Hambraeus L. (2000). Rates of Urea Production and Hydrolysis and Leucine Oxidation Change Linearly over Widely Varying Protein Intakes in Healthy Adults. J. Nutr..

[31] Ney D.M., Etzel M.R. (2017). Designing Medical Foods for Inherited Metabolic Disorders: Why Intact Protein Is Superior to Amino Acids. Curr. Opin. Biotechnol..

[32] Thiele A.G., Gausche R., Lindenberg C., Beger C., Arelin M., Rohde C., Mütze U., Weigel J.F., Mohnike K., Baerwald C. (2017). Growth and Final Height Among Children With Phenylketonuria. Pediatrics.

[33] Pinto A., Almeida M.F., MacDonald A., Ramos P.C., Rocha S., Guimas A., Ribeiro R., Martins E., Bandeira A., Jackson R. (2019). Over Restriction of Dietary Protein Allowance: The Importance of Ongoing Reassessment of Natural Protein Tolerance in Phenylketonuria. Nutrients.

[34] Sawin E.A., De Wolfe T.J., Aktas B., Stroup B.M., Murali S.G., Steele J.L., Ney D.M. (2015). Glycomacropeptide Is a Prebiotic That Reduces Desulfovibrio Bacteria, Increases Cecal Short-Chain Fatty Acids, and Is Anti-Inflammatory in Mice. Am. J. Physiol. Gastrointest. Liver Physiol..

[35] Montanari C., Ceccarani C., Corsello A., Zuvadelli J., Ottaviano E., Dei Cas M., Banderali G., Zuccotti G., Borghi E., Verduci E. (2022). Glycomacropeptide Safety and Its Effect on Gut Microbiota in Patients with Phenylketonuria: A Pilot Study. Nutrients.

[36] Jäger R., Purpura M., Farmer S., Cash H.A., Keller D. (2018). Probiotic Bacillus Coagulans GBI-30, 6086 Improves Protein Absorption and Utilization. Probiotics Antimicrob. Proteins.

[37] Martina A., Felis G.E., Corradi M., Maffeis C., Torriani S., Venema K. (2019). Effects of Functional Pasta Ingredients on Different Gut Microbiota as Revealed by TIM-2 in Vitro Model of the Proximal Colon. Benef. Microbes.

[38] Gill S.K., Rossi M., Bajka B., Whelan K. (2021). Dietary fibre in gastrointestinal health and disease. Nat. Rev. Gastroenterol. Hepatol..

[39] Pinto A., Almeida M.F., Ramos P.C., Rocha S., Guimas A., Ribeiro R., Martins E., Bandeira A., MacDonald A., Rocha J.C. (2017). Nutritional Status in Patients with Phenylketonuria Using Glycomacropeptide as Their Major Protein Source. Eur. J. Clin. Nutr..

[40] MacLeod E.L., Clayton M.K., van Calcar S.C., Ney D.M. (2010). Breakfast with Glycomacropeptide Compared with Amino Acids Suppresses Plasma Ghrelin Levels in Individuals with Phenylketonuria. Mol. Genet. Metab..

[41] Lammi C., Bollati C., Fiori L., Li J., Fanzaga M., d’Adduzio L., Tosi M., Burlina A., Zuccotti G., Verduci E. (2023). Glycomacropeptide (GMP) Rescued the Oxidative and Inflammatory Activity of Free L-AAs in Human Caco-2 Cells: New Insights That Support GMP as a Valid and Health-Promoting Product for the Dietary Management of Phenylketonuria (PKU) Patients. Food Res. Int..

[42] Matalon R., Michals-Matalon K., Bhatia G., Grechanina E., Novikov P., McDonald J.D., Grady J., Tyring S.K., Guttler F. (2006). Large neutral amino acids in the treatment of phenylketonuria (PKU). J. Inherit. Metab. Dis..

[43] Daly A., Högler W., Crabtree N., Shaw N., Evans S., Pinto A., Jackson R., Ashmore C., Rocha J.C., Strauss B.J. (2021). A Three-Year Longitudinal Study Comparing Bone Mass, Density, and Geometry Measured by DXA, pQCT, and Bone Turnover Markers in Children with PKU Taking L-Amino Acid or Glycomacropeptide Protein Substitutes. Nutrients.

[44] Hansen K.E., Murali S., Chaves I.Z., Suen G., Ney D.M. (2023). Glycomacropeptide Impacts Amylin-Mediated Satiety, Postprandial Markers of Glucose Homeostasis, and the Fecal Microbiome in Obese Postmenopausal Women. J. Nutr..

[45] Foisy Sauvé M., Spahis S., Delvin E., Levy E. (2021). Glycomacropeptide: A Bioactive Milk Derivative to Alleviate Metabolic Syndrome Outcomes. Antioxid. Redox Signal..

