# Supplementary material for: Glycomacropeptide-Based Protein Substitutes for Children with Phenylketonuria in Italy: A Nutritional Comparison
Source: Nutrients. 2024 Mar 27;16(7):956. doi: 10.3390/nu16070956 (PMC11013192; doi:10.3390/nu16070956)
Supplement: Supplementary file 1 [file nutrients-16-00956-s001.zip › nutrients-2910487-supplementary.pdf]

Table S1. Food components contained in 100 g of GMP-based protein substitute – powdered. If nutritional data was not present nutritional label, the term "Not Declared" (ND) was used.

Content per 100 g	Unit	MEDIFOOD		NUTRICIA	MAMOXI			MEVALIA		VITAFLO	
		powdered 1	powdered 2	powdered 3	powdered 4	powdered 5	powdered 6	powdered 7	powdered 8	powdered 9	powdered 10
Energy	Kj	1342	1336	1616	1257	1257	1257	1352	1338	1432	1432
Energy	Kcal	318	316	384	298	298	298	319	320	338	338

Content per 100 g	Unit	CAMBROOKE										
		powdered 11	powdered 12	powdered 13	powdered 14	powdered 15	powdered 16	powdered 17	powdered 18	powdered 19	powdered 20	powdered 21
Energy	Kj	1675	1674	1674	1400	1400	1439	1485	1395	1475	1527	1527
Energy	Kcal	400	385	400	335	335	344	355	333	353	365	365

Content per 100 g	Unit	MEDIFOOD		NUTRICIA	MAMOXI			MEVALIA		VITAFLO	
		powdered 1	powdered 2	powdered 3	powdered 4	powdered 5	powdered 6	powdered 7	powdered 8	powdered 9	powdered 10
Total fats	g	0.20	0.20	11.70	2.00	2.00	2.00	1.70	1.50	4.70	4.70
Saturated fatty acids	g	0.10	0.10	2.80	1.00	1.00	1.00	0.70	0.40	1.00	1.00
Monounsaturated fatty acids	g	ND	ND	6.40	0.80	0.80	0.80	ND	ND	0.50	0.50
Polyunsaturated fatty acids	g	ND	ND	2.50	0.30	0.30	0.30	ND	ND	1.20	1.20
Decosahexaenoic acid (DHA)	mg	ND	ND	155.00	ND	ND	ND	290.00	278.00	314.00	314.00
Eicosapentaenoic acid	mg	ND	ND	5.00	ND	ND	ND	64.00	62.00	ND	ND
Carbohydrates	g	39.10	38.80	37.50	8.00	8.00	8.00	32.00	33.00	18.00	18.00

Sugars	g	2.40	2.40	26.00	0.80	0.80	0.80	19.00	22.00	6.30 (8.00$)	6.30
Maltose	g	ND	ND	2.00	ND	ND	ND	ND	ND	ND	ND
Sucrose	g	ND	ND	24.00	ND	ND	ND	ND	ND	ND	ND
Fiber	g	ND	ND	4.50	14.00	14.00	14.00	3.00	3.00	ND	ND
Protein equivalents	g	40.00	40.00	30.00	55.00	55.00	55.00	43.00	41.00	56.00	56.00

**Content per 100 g**	**Unit**	**CAMBROOKE**										
		**powdered 11**	**powdered 12**	**powdered 13**	**powdered 14**	**powdered 15**	**powdered 16**	**powdered 17**	**powdered 18**	**powdered 19**	**powdered 20**	**powdered 21**
Total fats	g	11.00	11.50	11.00	4.20	4.20	6.30	6.50	6.10	6.40	0	0
Saturated fatty acids	g	4.60	4.50	4.50	0.70	0.70	0.80	0.70	0.60	0.70	0	0
Monounsaturated fatty acids	g	2.40	2.30	2.30	ND	ND	3.20	2.70	3.80	2.80	ND	ND
Polyunsaturated fatty acids	g	4.20	4.00	4.00	ND	ND	1.50	1.50	1.50	1.50	ND	ND
Decosahexaenoic acid (DHA)	mg	192.00	62.0	62.00	167.00	167.00	156.00	161.00	152.00	160.00	ND	ND
Eicosapentaenoic acid	mg	ND	ND	ND	ND	ND	ND	ND	ND	ND	ND	ND
Carbohydrates	g	37.00	50.00	50.00	2.40	2.40	5.20	5.50	5.50	5.40	68.00	68.00
Sugars	g	12.00	13.00	13.00	0.80	0.80	0.60	0.80	0.80	0.60	65.00	65.00
Maltose	g	ND	ND	ND	ND	ND	ND	ND	ND	ND	ND	ND
Sucrose	g	ND	ND	ND	ND	ND	ND	ND	ND	ND	ND	ND
Fiber	g	0	0.10	0	0	0	1	1.00	1.00	1.00	0	0

Protein equivalents	g	38.00	38.00	38.00	67.00	67.00	63.00	64.00	64.00	64.00	25.00	25.00

Content per 100 g	Unit	MEDIFOOD		NUTRICIA	MAMOXI			MEVALIA		VITAFLO	
		**powdered 1**	**powdered 2**	**powdered 3**	**powdered 4**	**powdered 5**	**powdered 6**	**powdered 7**	**powdered 8**	**powdered 9**	**powdered 10**
L-Alanine	g	1.82	1.82	1.20	5.80	5.80	5.80	2.76	2.65	2.36	2.23
L-Arginine	g	2.17	2.17	1.50	2.00	2.00	2.00	2.02	1.89	2.73	2.71
L-Aspartic acid	g	2.65	2.65	1.80	5.40	5.40	5.40	4.80	4.45	3.73	3.2
L-Cystine	mg	25.23	25.23	870.00	30.00	30.00	30.00	250	0.21	0.00	0.00
L-Glutamic acid	g	5.98	5.98	4.00	6.00	6.00	6.00	4.17 (L-glutamine 2.76)	3.74 (L-glutamine 2.65)	7.72	7.34
Glycine	g	0.33	0.33	0.20	8.40	8.40	8.40	3.36	3.24	2.02	3.43
L-Histidine	g	1.63	1.63	1.20	1.20	1.20	1.20	1.28	1.22	2.01	2.00
L-Isoleucine	g	3.05	3.05	2.20	3.30	3.30	3.30	1.84	1.83	4.06	3.86
L-Leucine	g	5.17	5.17	4.50	5.00	5.00	5.00	2.65	2.56	8.63	8.57
L-Lysine	g	3.11	3.11	1.30	3.10	3.10	3.10	3.47	3.24	2.72	2.29
L-Methionine	g	0.56	0.56	0.40	0.60	0.60	0.60	0.79	0.80	0.80	0.80
L-Phenylalanine	mg	50.46	50.46	54.00	91.00	91.00	91.00	61.00	61.00	104.00	104.00
L-Proline	g	3.51	3.51	2.40	3.70	3.70	3.70	5.39	5.17	4.58	4.34
L-Serine	g	2.27	2.27	1.50	2.40	2.40	2.40	1.42	1.31	2.88	2.74
Taurine	mg	ND	ND	90.00	54.00	54.00	54.00	ND	ND	ND	ND
L-Threonine	g	5.07	5.07	3.40	5.30	5.30	5.30	4.03	3.87	6.55	6.29

L-Tryptophan	g	0.86	0.86	0.72	0.90	0.90	0.90	0.77	0.71	1.15	1.14
L-Tyrosine	g	5.73	5.73	3.00	5.80	5.80	5.80	6.63	6.31	6.43	6.4
L-Valine	g	3.25	3.25	2.60	2.70	2.70	2.70	1.48	1.47	3.27	3.11
L-Carnitine	mg	ND	ND	43.20	49.00	49.00	49.00	25.00	24.00	ND	ND

Content per 100 g	Unit	CAMBROOKE										
		**powdered 11**	**powdered 12**	**powdered 13**	**powdered 14**	**powdered 15**	**powdered 16**	**powdered 17**	**powdered 18**	**powdered 19**	**powdered 20**	**powdered 21**
L-Alanine	g	0.94	0.95	0.9	2.26	2.26	2.10	2.17	2.17	2.16	1.07	1.07
L-Arginine	g	3.12	3.12	3.12	7.06	7.06	6.56	6.77	6.77	6.73	2.61	2.61
L-Aspartic acid	g	1.26	1.26	1.26	3.00	3.00	2.78	2.78	2.78	2.85	1.54	1.54
L-Cystine	mg	15.00	15.00	15.0	33.00	33.00	34.00	35.00	35.00	35.00	20.00	20.00
L-Glutamic acid	g	3.06	3.06	3.06	7.33	7.33	6.81	7.03	7.03	6.98	3.61	3.61
Glycine	g	2.00	2.00	2.00	3.06	3.06	2.84	2.93	2.93	2.91	0.19	0.19
L-Histidine	g	0.70	0.70	0.70	1.60	1.60	1.48	1.53	1.53	1.52	0.63	0.63
L-Isoleucine	g	1.61	1.61	1.61	3.88	3.86	3.56	3.67	3.67	3.65	1.82	1.82
L-Leucine	g	3.22	3.22	3.22	7.66	7.66	7.10	7.33	7.33	7.29	5.29	5.29
L-Lysine	g	2.73	2.73	2.73	4.00	4.00	3.71	3.83	3.83	3.81	1.09	1.09
L-Methionine	g	0.36	0.37	0.37	0.67	0.67	0.61	0.63	0.63	0.62	0.28	0.28
L-Phenylalanine	mg	38.00	31.00	31.00	67.00	67.00	62.00	65.00	65.00	64.00	45.00	45.00
L-Proline	g	1.77	1.77	1.77	4.26	4.26	3.93	4.06	4.06	4.03	2.03	2.03
L-Serine	g	1.00	1.00	1.00	2.40	2.40	2.21	2.29	2.29	2.27	1.15	1.15

Taurine	mg	ND	ND	ND	ND	ND	ND	ND	ND	ND	ND	ND
L-Threonine	g	2.51	2.51	2.51	6.00	6.00	5.57	5.75	5.75	5.72	2.83	2.83
L-Tryptophan	g	776.00	776.00	776.00	0.93	0.93	0.86	0.89	0.89	0.89	0.34	0.34
L-Tyrosine	g	2.92	2.38	2.92	6.20	6.20	5.75	5.93	5.93	5.89	0.66	0.66
L-Valine	g	1.38	1.38	1.38	3.30	3.30	3.06	3.16	3.16	3.14	1.49	1.49
L-Carnitine	mg	ND	ND	ND	ND	ND	ND	ND	ND	ND	ND	ND

Content per 100 g	Unit	MEDIFOOD		NUTRICIA	MAMOXI			MEVALIA		VITAFLO	
		powdered 1	powdered 2	powdered 3	powdered 4	powdered 5	powdered 6	powdered 7	powdered 8	powdered 9	powdered 10
Vitamin A	µg RE	555.00	555.00	500.00	974.00	974.00	974.00	1248.00	1198.00	740.00	740.00
Vitamin C	mg	72.00	72.00	72.00	114.00	114.00	114.00	187.00	180.00	74.00	74.00
Vitamin D	µg	8.80	8.80	22.50	22.00	22.00	22.00	37.00	36.00	14.00	14.00
Vitamin E	mg α-TE	10.40	10.40	15.30	19.00	19.00	19.00	25.00	24.00	15.00	15.00
Vitamin K	µg	67.00	67.00	72.00	70.00	70.00	70.00	75.00	72.00	67.00	67.00
Vitamin B1. Thiamin	mg	1.30	1.30	1.80	2.00	2.00	2.00	2.5.00	2.40	1.70	1.70
Vitamin B2. Riboflavin	mg	1.50	1.50	1.80	2.00	2.00	2.00	3.12	3.00	1.70	1.70
Vitamin B3. Niacin	mg	16.50	16.50	15.00	43.00	43.00	43.00	15.00	14.00	9.10	9.10
Niacin (NE)	mg	ND	ND	ND	58.00	58.00	58.00	ND	ND	28.00	28.00
Vitamin B6	mg	1.90	1.90	1.70	1.70	1.70	1.70	2.50	2.40	1.70	1.70
Total folate	µg	267.00	267.00	360.00	217.00	217.00	217.00	312	300.00	290.00	290.00
Vitamin B12	µg	3.20	3.20	3.60	3.50	3.50	3.50	5.00	4.79	4.60	4.60

Pantotenic acid	mg	5.00	5.00	4.80	7.00	7.00	7.00	8.74	8.39	5.70	5.70
Biotin	µg	125.00	125.00	36.00	44.00	44.00	44.00	75.00	72.00	36.00	36.00
Choline	mg	400.00	400.00	300.00	486.00	486.00	486.00	438.00	420.00	570.00	570.00
Calcium	mg	797.00	797.00	1155.00	1514.00	1514.00	1514.00	1774.00	1703.00	1140.00	1140.00
Chromium	µg	59.00	59.00	39.60	108.00	108.00	108.00	50.00	48.00	34.00	34.00
Copper	mg	1.49	1.49	1.30	2.00	2.00	2.00	1.31	1.26	1.70	1.70
Iodine	µg	168.00	168.00	149.00	244.00	244.00	244	276.00	264.00	240.00	240.00
Iron	mg	14.40	14.40	14.50	19.00	19.00	19.00	25.00	21.00	21.00	21.00
Magnesium	mg	251.00	251.00	277.00	292.00	292.00	292	329.00	294.00	330.00	330.00
Manganese	mg	2.05	2.05	1.80	6.00	6.00	6.00	1.31	1.26	1.10	1.10
Molybdenum	µg	96.00	96.00	59.40	92.00	92.00	92.00	73.00	71.00	57.00	57.00
Phosphorus	mg	851.00	851.00	1040.00	935.00	935.00	935.00	1391.00	1256.00	1180.00	1180.00
Selenium	µg	59.00	59.00	52.80	79.00	79.00	79.00	62.00	60.00	86.00	86.00
Zinc	mg	14.40	14.40	11.20	19.00	19.00	19.00	13.00	13.00	21.00	21.00
Potassium	mg	627.00	627.00	1152.00	789.00	789.00	789.00	1753.00	1683.00	670.00	670.00
Sodium	mg	339.00	339.00	512.00	606.00	673.00	674.00	329.00	315.00	810.00	810.00
Chloride	mg	670.00	670.00	626.00	38.00	137.00	38.00	<20	<20	20.00	20.00
Fluoride	mg	ND	ND	ND	0.80	0.80	0.80	ND	ND	ND	ND
Myo-inositol	mg	ND	ND	ND	254	254	254.00	ND	ND	ND	ND
Salt	g	0.85	0.85	1.30	1.50	1.50	1.50	0.82	0.96	2.00	2.00

Content per 100 g	Unit	CAMBROOKE										
		powdered 11	powdered 12	powdered 13	powdered 14	powdered 15	powdered 16	powdered 17	powdered 18	powdered 19	powdered 20	powdered 21
Vitamin A	μg RE	707.00	707.00	707.00	1098.00	1098.00	1019.00	1052.00	1052.00	1045.00	0.00	0.00
Vitamin C	mg	81.00	81.00	81.00	177.00	177.00	166.00	171.00	171.00	170.00	0.00	0.00
Vitamin D	μg	35.00	35.00	35.00	48.00	48.00	44.00	45.00	45.00	45.00	0.00	0.00
Vitamin E	mg α-TE	12.00	12.00	12.00	20.00	20.00	19.00	19.00	19.00	18.00	0.00	0.00
Vitamin K	μg	80.00	80.00	80.00	60.00	60.00	112.00	114.00	114.00	114.00	0.00	0.00
Vitamin B1. Thiamin	mg	1.50	1.50	1.50	60.00	60.00	1.50	1.60	1.60	1.60	0.00	0.00
Vitamin B2. Riboflavin	mg	1.50	1.50	1.50	1.70	1.70	1.50	1.60	1.60	1.60	1.60	1.60
Vitamin B3. Niacin	mg	28.00	28.00	28.00	1.70	1.70	33.00	34.00	34.00	34.00	20.00	20.00
Niacin (NE)	mg	ND	ND	ND	ND	ND	ND	ND	ND	ND		
Vitamin B6	mg	1.50	1.50	1.50	1.70	1.70	1.60	1.60	1.60	1.60	2.00	2.00
Total folate	μg	199.00 (337.00 ugDFE)	199.00	288.00 (337.00 ugDFE)	240.00	240.00	219.00	226.00	226.00	224.00	400.00	400.00
Vitamin B12	μg	2.80	2.80	2.80	3.00	3.00	2.80	2.90	2.90	2.90	6.00	6.00
Pantotenic acid	mg	3.10	5.60	5.60	7.30	7.30	6.80	7.00	7.00	7.10	10.00	10.00
Biotin	μg	24.00	24.00	24.00	32.00	32.00	30.00	31.00	31.00	30.00	300.00	300.00
Choline	mg	375.00	375.00	375.00	99.00	99.00	92.00	95.00	95.00	95.00	0.00	0.00
Calcium	mg	1353.00	1353.00	1354.00	1774.00	1774.00	1668.00	1742.00	1742.00	1731.00	20.00	20.00
Chromium	μg	33.00	25.00	33.00	52.00	52.00	50.00	52.00	52.00	51.00	0.00	0.00
Copper	mg	0.94	0.94	0.94	0.70	0.70	0.60	0.60	0.60	0.60	0.00	0.00
Iodine	μg	163.00	163.00	163.00	229.00	229.00	213.00	219.00	219.00	218.00	0.00	0.00
Iron	mg	16.00	16.00	16.00	0.03	0.03	0.02	0.03	0.03	0.03	0.00	0.00
Magnesium	mg	168.00	168.00	168.00	327.00	327.00	303.00	313.00	313.00	311.00	18.00	18.00

Manganese	mg	2.30	2.30	2.30	0.00	0.00	0.00	0.00	0.00	0.00	0.00	0.00
Molybdenum	µg	46.00	46.00	46.00	60.00	60.00	56.00	58.00	58.00	0.06	0.00	0.00
Phosphorus	mg	1089.00	1089.00	1089.00	1676.00	1676.00	1556	1606.00	1606.00	1596.00	100.00	100.00
Selenium	µg	45.00	45.00	45.00	80.00	80.00	74.00	77.00	77.00	76.00	0.00	0.00
Zinc	mg	7.50	7.50	7.50	15.00	15.00	14.00	15.00	15.00	14.00	0.00	0.00
Potassium	mg	1228.00	1240.00	1241.00	1822.00	1822.00	1750.00	1806.00	1806.00	1795.00	645.00	655.00
Sodium	mg	726.00	472.00	471.00	881.00	881.00	844.00	871.00	871.00	865.00	890.00	835.00
Chloride	mg	583.00	179.00	189.00	732.00	732.00	719.00	742.00	742.00	737.00	245.00	245.00
Fluoride	mg	ND	ND	ND	ND	ND	ND	ND	ND	ND	ND	ND
Myo-inositol	mg	ND	ND	ND	ND	ND	ND	ND	ND	ND	ND	ND
Salt	g	1.80	1.20	1.20	2.20	2.20	2.10	2.20	2.20	2.20	2.00	2.00

Explanation of GMP-based protein substitutes – powdered.

MEDIFOOD. GMP-based powdered 1: Afenil GMP Up Shake milk; GMP-based powdered 2: Afenil GMP Up Shake orange. Recommended serving of 25 g.

NUTRICIA. GMP-based powdered 3: PKU GMPro. Recommended serving of 33,3 g.

MAMOXI. GMP-based powdered 4: XPhe Enjoy GMP Fibre; GMP-based powdered 5: XPhe Enjoy GMP Chocolate Fibre; GMP-based powdered 6: XPhe Enjoy GMP Vanilla Fibre. Recommended serving of 18,5 g or 37 g.

MEVALIA. GMP-based powdered 7: PKU GMP Power; GMP-based powdered 8: PKU GMP Power Pina Colada. Recommended serving of 24,4 g.

VITAFLO. GMP-based powdered 9: PKU Sphere 15; $ PKU Sphere 15 Chocolate. Recommended serving of 27 g. GMP-based powdered 10: PKU Sphere 20. Recommended serving of 35 g.

CAMBROOKE. GMP-based powdered 11: Glytactin Bettermilk 15; GMP-based powdered 12: Glytactin Bettermilk 15 Orange; GMP-based powdered 13: Glytactin Bettermilk 15 Strawberry; Recommended serving of 40 g. GMP-based powdered 14: Glytactin Build 10; Recommended serving of 15 g. GMP-based powdered 15: Glytactin Build 20; Recommended serving of 30 g. GMP-based powdered 16: Glytactin Build 20 Chocolate; Recommended serving of 32 g. GMP-based powdered 17: Glytactin Build 20 Raspberry Lemonade; Recommended serving of 31 g. GMP-based powdered 18: Glytactin Build 20 Vanilla; Recommended serving of 33 g. GMP-based powdered 19: Glytactin Build 20 Smooth; Recommended serving of 31 g. GMP-based powdered 20: Glytactin Restore Powder Orange; GMP-based powdered 21: Glytactin Restore Powder Berry; Recommended serving of 20 g.

Table S2. Food components contained in 100 ml of GMP-based protein substitute – ready to drink. If nutritional data was not present nutritional label, the term "Not Declared" (ND) was used.

Content per 100 ml	Unit	NUTRICIA	CAMBROOKE						
		ready to drink 1	ready to drink 2	ready to drink 3	ready to drink 4	ready to drink 5	ready to drink 6	ready to drink 7	ready to drink 8
Energy	Kj	188	272.8	272.8	346	346	201	201	59
Energy	Kcal	45	64.6	64.6	82	82	48	48	14

Content per 100 ml	Unit	NUTRICIA	CAMBROOKE						
		ready to drink 1	ready to drink 2	ready to drink 3	ready to drink 4	ready to drink 5	ready to drink 6	ready to drink 7	ready to drink 8
Total fats	g	1.60	1.40	1.40	2.00	2.00	1.40	1.40	0.00
Saturated fatty acids	g	0.16	0.40	0.40	0.80	0.80	0.20	0.20	0.00
Monounsaturated fatty acids	g	0.95	ND	ND	ND	ND	ND	ND	ND
Polyunsaturated fatty acids	g	0.49	ND	ND	ND	ND	ND	ND	ND
Decosahexaenoic acid (DHA)	mg	24.00	ND	ND	ND	ND	44.00	44.00	ND
Eicosapentaenoic acid	mg	6.00	ND	ND	ND	ND	ND	ND	ND
Carbohydrates	g	3.40	9.00	9.00	10.00	10.00	2.90	3.00	1.40
Sugars	g	3.00	7.00	7.00	8.00	8.00	0.00	0.00	1.00
Fiber	g	0.37	0.40	0.00	0.40	0.40	0.90	0.90	0.00
Protein equivalents	g	4.00	4.00	4.00	6.00	6.00	6.00	6.00	2.00

Content per 100 ml	Unit	NUTRICIA	CAMBROOKE						
		ready to drink 1	ready to drink 2	ready to drink 3	ready to drink 4	ready to drink 5	ready to drink 6	ready to drink 7	ready to drink 8
L-Alanine	g	0.16	0.14	0.14	0.22	0.22	0.23	0.23	0.10

L-Arginine	g	0.19	0.41	0.41	0.62	0.62	0.60	0.60	0.22
L-Aspartic acid	g	0.24	0.22	0.22	0.32	0.32	0.30	0.30	0.14
L-Cystine	g	0.12	0.00	0.00	0.00	0.00	0.00	0.00	0.00
L-Glutamic acid	g	0.54	0.52	0.52	0.80	0.80	0.74	0.74	0.33
Glycine	g	0.03	0.30	0.03	0.42	0.04	0.04	0.04	0.01
L-Histidine	g	0.16	0.87	0.87	0.13	0.13	0.14	0.14	0.05
L-Isoleucine	g	0.29	0.25	0.25	0.38	0.38	0.39	0.39	0.18
L-Leucine	g	0.61	0.79	0.79	1.20	1.20	1.08	1.08	0.42
L-Lysine	g	0.17	0.17	0.17	0.26	0.26	0.36	0.36	0.10
L-Methionine	g	0.05	0.04	0.04	0.60	0.06	0.06	0.67	0.03
L-Phenylalanine	mg	7.20	7.20	7.20	10.80	10.80	6.00	6.00	3.00
L-Proline	g	0.32	0.34	0.34	0.51	0.51	0.43	0.43	0.20
L-Serine	g	0.20	0.14	0.14	0.22	0.22	0.24	0.24	0.13
Taurine	mg	12.00	ND	ND	ND	ND	ND	ND	ND
L-Threonine	g	0.45	0.43	0.43	0.64	0.64	0.61	0.61	0.22
L-Tryptophan	g	0.10	0.06	0.62	0.93	0.93	0.08	0.84	0.03
L-Tyrosine	g	0.40	0.30	0.30	0.46	0.46	0.55	0.55	0.06
L-Valine	g	0.35	0.22	0.22	0.33	0.33	0.33	0.33	0.15
L-Carnitine	mg	5.80	ND	ND	ND	ND	ND	ND	ND

**Content per 100 ml**	**Unit**	**NUTRICIA**	**CAMBROOKE**						
		**ready to drink 1**	**ready to drink 2**	**ready to drink 3**	**ready to drink 4**	**ready to drink 5**	**ready to drink 6**	**ready to drink 7**	**ready to drink 8**
Vitamin A	µg RE	80.00	102.00	102.00	108.00	108.00	108.00	108.00	ND
Vitamin C	mg	6.20	8.00	8.00	11.20	11.20	11.00	11.00	ND

Vitamin D	μg	2.50	2.50	2.50	2.50	2.50	2.50	2.50	ND
Vitamin E	mg α-TE	1.30	0.90	0.90	0.90	0.90	0.90	0.90	ND
Vitamin K	μg	7.00	12.00	12.00	12.00	12.00	12.00	12.00	ND
Vitamin B1. Thiamin	mg	0.16	0.20	0.20	0.20	0.20	0.20	0.20	ND
Vitamin B2. Riboflavin	mg	0.16	0.20	0.20	0.20	0.20	0.20	0.20	0.10
Vitamin B3. Niacin	mg	1.40	1.60	1.60	2.00	2.00	2.00	2.00	1.00
Vitamin B6	mg	0.14	0.20	0.20	0.20	0.20	0.20	0.20	0.10
Total folate	μg	20.00	53.20	53.20	56.00	56.00	24.00	24.00	20.00
Vitamin B12	μg	0.28	0.30	0.30	0.30	0.30	0.30	0.30	0.40
Pantotenic acid	mg	0.52	0.70	0.70	0.70	0.70	0.70	0.70	0.50
Biotin	μg	3.10	3.20	3.20	3.20	3.20	3.20	3.20	15.00
Choline	mg	37.50	81.20	81.20	82.40	82.40	80.00	80.00	ND
Calcium	mg	144.00	130.00	130.00	140.00	140.00	141.00	141.00	2.00
Chromium	μg	3.30	4.00	4.00	5.20	5.20	5.20	5.20	ND
Copper	mg	0.10	0.10	0.10	0.10	0.10	0.10	0.10	ND
Iodine	μg	16.20	22.80	22.80	22.80	22.80	23.00	23.00	ND
Iron	mg	1.40	1.60	1.60	1.80	1.80	2.00	2.00	ND
Magnesium	mg	26.00	30.80	30.80	48.00	48.00	54.00	54.00	1.30
Manganese	mg	0.10	0.30	0.30	0.30	0.30	0.30	0.30	ND
Molybdenum	μg	5.50	5.60	5.60	6.00	6.00	6.00	6.00	ND
Phosphorus	mg	121.00	126.00	126.00	126.00	126.00	125.00	125.00	8.00
Selenium	μg	5.50	8.20	8.20	8.00	8.00	8.00	8.00	ND
Zinc	mg	0.96	1.30	1.30	1.30	1.30	1.30	1.30	ND
Potassium	mg	82.40	136.00	136.00	136	136.00	141.00	141.00	44.00

Sodium	mg	8.10	100.00	100.00	96.00	96.00	114.00	114.00	76.00
Chloride	mg	44.00	64.00	64.00	64.80	64.80	116.00	116.00	1.60
Salt	g	0.16	0.25	0.25	0.24	0.24	0.30	0.30	0.19

Explanation of GMP-based protein substitutes – ready to drink.

NUTRICIA. GMP-based ready to drink 1: PKU GMPro LQ.

CAMBROOKE. GMP-based ready to drink 2: Glytactin RTD 10 smooth; GMP-based ready to drink 3: Glytactin RTD 10 chocolate; GMP-based ready to drink 4: Glytactin RTD 15 smooth; GMP-based ready to drink 5: Glytactin RTD 15 chocolate; GMP-based ready to drink 6: Glytactin RTD 15 lite mocha; GMP-based ready to drink 7: Glytactin RTD 15 lite vanilla; GMP-based ready to drink 8: Glytactin restore lite 10 Tangerine.

All recommended servings are of 250 ml, apart from GMP-based ready to drink 8 whose recommended serving is of 480 ml.

Table S3. Food components contained in 100 g and per portion of GMP-based protein substitute - bar. If nutritional data was not present nutritional label, the term "Not Declared" (ND) was used.

Content per 100 g and per portion	Unit	MEDIFOOD				CAMBROOKE							
		bar 1 (50 g)	bar 1 (100 g)	bar 2 (60 g)	bar 2 (100 g)	bar 3 (54 g)	bar 3 (100 g)	bar 4 (81 g)	bar 4 (100 g)	bar 5 (54 g)	bar 5 (100 g)	bar 6 (81 g)	bar 6 (100 g)
Energy	Kj	661	1322	884	1473	878.60	1627.6	1338.9	1652.7	920.5	1702.9	1380.7	1702.9
Energy	Kcal	156	313	210	349	210	389	320	395	220	407	330	407

Content per 100 g and per portion	Unit	MEDIFOOD				CAMBROOKE							
		bar 1 (50 g)	bar 1 (100 g)	bar 2 (60 g)	bar 2 (100 g)	bar 3 (54 g)	bar 3 (100 g)	bar 4 (81 g)	bar 4 (100 g)	bar 5 (54 g)	bar 5 (100 g)	bar 6 (81 g)	bar 6 (100 g)
Total fats	g	0.90	1.80	4.30	7.20	6.00	11.10	8.00	9.90	8.00	14.80	12.00	14.80
Saturated fatty acids	g	0.40	0.80	3.40	5.70	4.00	7.40	6.00	7.40	7.00	13.00	9.00	11.10
Carbohydrates	g	24.70	50.00	30.20	50.00	30.00	56.00	45.00	56.00	26.00	48.00	34.00	47.00
Sugars	g	6.20	12.40	12.00	20.00	14.00	26.00	19.00	23.00	19.00	35.00	25.00	31.00
Polyols	g	ND	ND	ND	ND	5.00	9.00	7.00	9.00	1.00	2.00	1.00	1.00
Fiber	g	5.00	8.40	5.00	8.40	2.00	4.00	3.00	4.00	2.00	4.00	3.00	4.00
Protein equivalents	g	10.00	20.00	10.00	16.70	10.00	19.00	15.00	19.00	10.00	19.00	15.00	19.00

Content per 100 g and per portion	Unit	MEDIFOOD				CAMBROOKE							
		bar 1 (50 g)	bar 1 (100 g)	bar 2 (60 g)	bar 2 (100 g)	bar 3 (54 g)	bar 3 (100 g)	bar 4 (81 g)	bar 4 (100 g)	bar 5 (54 g)	bar 5 (100 g)	bar 6 (81 g)	bar 6 (100 g)
L-Alanine	g	0.45	0.90	0.45	0.75	0.40	0.74	0.58	0.71	0.40	0.74	0.60	0.74
L-Arginine	g	0.54	1.08	0.54	0.90	1.10	2.03	1.60	1.97	1.00	1.85	1.30	1.60
L-Aspartic acid	g	0.66	1.32	0.66	1.10	0.60	1.11	0.88	1.08	0.50	0.92	0.88	1.08
L-Cystine	g	0.66	12.62	6.31	10.52	0.00	0.00	0.02	0.02	0.00	0.00	0.02	0.24
L-Glutamine	g	1.50	3.00	1.50	2.50	1.30	2.40	1.90	2.34	1.20	2.22	2.10	2.59
Glycine	g	0.08	0.16	0.08	0.13	0.10	0.18	0.12	0.14	0.10	0.18	0.12	0.14
L-	g	0.41	0.82	0.41	0.68	0.30	0.55	0.38	0.46	0.20	0.37	0.38	0.46

Histidine													
L-Isoleucine	g	0.76	1.52	0.76	1.27	0.69	1.27	1.04	1.28	0.66	1.22	0.99	1.22
L-Leucine	g	1.29	2.58	1.29	2.15	2.03	3.75	3.04	3.75	1.92	3.55	2.88	3.55
L-Lysine	g	0.78	1.56	0.78	1.30	0.40	0.74	0.58	0.71	0.40	0.74	0.59	0.72
L-Methionine	g	0.14	0.28	0.14	0.23	0.10	0.18	0.15	0.18	0.10	0.18	0.15	0.18
L-Phenylalanine	mg	12.62	25.24	12.62	21.03	24.00	44.4	37.00	45.7	22.00	40.70	32.00	39.5
L-Proline	g	0.88	1.76	0.88	1.47	0.80	1.48	1.30	1.60	0.80	1.48	1.30	1.60
L-Serine	g	0.57	1.14	0.57	0.95	0.40	0.74	0.66	0.81	0.40	0.74	0.62	0.76
Taurine	mg	ND	ND	ND	ND	ND	ND	ND	ND	ND	ND	ND	ND
L-Threonine	g	1.27	2.54	1.27	2.12	1.10	2.03	1.62	2.00	1.00	1.85	1.51	1.86
L-Tryptophan	g	0.22	0.44	0.22	0.37	0.11	0.20	0.18	0.22	0.10	0.18	0.18	0.22
L-Tyrosine	g	1.43	2.86	1.43	2.38	0.86	1.59	1.30	1.60	0.84	1.55	1.26	1.55
L-Valine	g	0.81	1.62	0.81	1.35	0.59	1.09	0.90	1.11	0.59	1.09	0.89	1.09
L-Carnitine	mg	ND	ND	ND	ND	ND	ND	ND	ND	ND	ND	ND	ND
L-Glutamic acid	g	ND	ND	ND	ND	ND	ND	ND	ND	ND	ND	ND	ND

**Content per 100 g and per portion**	**Unit**	**MEDIFOOD**				**CAMBROOKE**							
		**bar 1 (50 g)**	**bar 1 (100 g)**	**bar 2 (60 g)**	**bar 2 (100 g)**	**bar 3 (54 g)**	**bar 3 (100 g)**	**bar 4 (81 g)**	**bar 4 (100 g)**	**bar 5 (54 g)**	**bar 5 (100 g)**	**bar 6 (81 g)**	**bar 6 (100 g)**
Vitamin A	µg RE	277.00	554.00	139.00	231.00	475.20	880.00	702.60	867.30	472.20	874.50	543.50	671.00
Vitamin C	mg	36.00	72.00	18.00	30.00	17.00	31.50	34.00	42.00	17.00	31.50	29.20	36.00
Vitamin D	µg	4.00	8.00	2.20	4.00	8.70	16.00	5.80	7.20	10.00	18.50	6.30	7.70
Vitamin E	mg α-TE	5.00	10.00	2.60	4.00	3.40	6.30	3.80	4.70	3.20	5.90	3.80	4.70
Vitamin K	µg	33.00	66.00	17.00	28.00	33.00	61.10	35.40	43.70	32.90	60.90	35.00	43.2
Vitamin B1. Thiamin	mg	0.67	1.34	0.30	0.60	0.40	0.70	0.50	0.60	0.40	0.70	0.50	0.60
Vitamin	mg	0.76	1.52	0.38	0.60	0.70	1.30	0.50	0.60	0.40	0.70	0.50	0.60

B2. Riboflavin													
Vitamin B3. Niacin	mg	8.00	16.00	4.10	7.00	5.60	10.40	6.10	7.50	5.80	10.70	6.10	7.50
Vitamin B6	mg	0.90	1.80	0.50	0.80	0.40	0.70	0.50	0.60	0.40	0.70	0.50	0.60
Total folate	μg	133.00	266.00	67.00	111.00	119.30	220.90	136.50	168.50	119.00	220.40	136.00	169.00
Vitamin B12	μg	2.00	4.00	0.80	1.00	0.80	1.50	0.90	1.10	0.80	1.50	0.90	1.10
Pantotenic acid	mg	2.70	5.40	1.30	2.20	1.80	3.30	1.70	2.10	1.80	3.30	1.70	2.10
Biotin	μg	63.00	126.00	31.00	52.00	7.20	13.30	8.50	10.50	7.20	13.30	8.50	10.5
Choline	mg	100.00	200.00	100.00	166.67	166.60	308.50	209.20	258.30	149.90	277.60	209.10	258.10
Calcium	mg	399.00	798.00	199.00	332.00	518.90	960.90	626.50	773.50	545.80	545.80	652.90	806.00
Chromium	μg	29.00	58.00	15.00	24.00	10.00	18.50	13.30	16.40	8.90	16.50	13.20	16.30
Copper	mg	747.00	1494.00	373.00	622.00	0.30	0.60	0.30	0.40	0.20	0.40	0.30	0.40
Iodine	μg	84.00	168.00	42.00	70.00	59.80	110.70	56.90	70.20	58.80	108.90	65.20	80.5
Iron	mg	7.00	14.00	3.60	6.00	7.70	14.30	5.80	7.20	6.50	12.00	5.90	7.30
Magnesium	mg	125.00	250.00	63.00	104.00	86.40	160.00	134.60	166.20	83.20	154.10	136.90	169.00
Manganese	mg	1.00	2.00	0.51	0.90	0.90	1.70	0.90	1.10	0.80	1.50	4.90	6.00
Molybdenum	μg	48.00	96.00	24.00	40.00	14.60	27.00	16.30	20.10	13.10	24.30	16.20	20.00
Phosphorus	mg	425.00	850.00	213.00	354.00	356.90	660.90	479.90	592.50	358.00	663.0	463.30	572.00
Selenium	μg	29.00	58.00	15.00	25.00	20.10	37.20	21.00	25.90	18.60	34.40	22.00	27.20
Zinc	mg	7.00	14.00	4.00	6.00	3.10	5.70	4.60	5.70	3.30	6.10	4.90	6.00
Potassium	mg	313.00	626.00	157.00	261.00	160.00	296.30	375.00	463.00	170.00	314.80	375.00	463.00
Sodium	mg	169.00	338.00	85.00	141.00	135.00	250.00	204.00	251.00	140.00	259.30	220.00	271.60
Chloride	mg	335.00	670.00	167.00	279.00	8.90	16.50	13.50	16.70	14.90	27.60	25.10	31.00
Salt	g	0.21	0.35	0.21	0.35	0.30	0.60	0.40	0.50	0.30	0.60	0.50	0.70

Explanation of GMP-based protein substitutes – bar.

MEDIFOOD. GMP-based bar 1: AFENIL GMP UP BAR CREAM-MOU; GMP-based bar 2: AFENIL GMP UP BAR COCONUT.

CAMBROOKE. GMP-based bar 3: Glytactin Complete cocoa 10; GMP-based bar 4: Glytactin Complete cocoa 15; GMP-based bar 5: Glytactin Complete fruit 10; GMP-based bar 5: Glytactin Complete fruit 15.

Table S4. Mean of the food components contained in 100 g of 3 different L-AAs powdered protein substitute available for children with PKU from 3 years of age. If nutritional data was not present nutritional label, the term "Not Declared" (ND) was used.

Content per 100 g	Unit	MEDIFOOD	MAMOXI	NUTRICIA	MEAN OF 3 L-AAs PS
Energy	Kj	1409	1291	1487	1395.7
Energy	Kcal	334	304	350	329.3

Content per 100 g	Unit	MEDIFOOD	MAMOXI	NUTRICIA	MEAN OF 3 L-AAs PS
Total fats	g	<0.5	<0.3	1.50	1.50*
Saturated fatty acids	g	<0.1	<0.1	0.52	0.52*
Monounsaturated fatty acids	g	ND	0.0	0.35	0.18*
Polyunsaturated fatty acids	g	ND	0.0	0.59	0.30*
Decosahexaenoic acid (DHA)	mg	ND	ND	540	540*
Eicosapentaenoic acid	mg	ND	ND	ND	ND
Carbohydrates	g	28.00	11.00	13.60	17.53
Sugars	g	2.90	9.00	0.10	4.00
Maltose	g	ND	ND	ND	ND
Sucrose	g	ND	ND	ND	ND
Fiber	g	21.00	1.00	0.83	7.61
Protein equivalents	g	45.00	63.00	71.40	59.80

Content per 100 g	Unit	MEDIFOOD	MAMOXI	NUTRICIA	MEAN OF 3 L-AAs PS
L-Alanine	g	1.81	3.40	3.71	2.97
L-Arginine	g	2.89	3.10	6.36	4.12
L-Aspartic acid	g	4.58	9.00	5.57	6.38
L-Cystine	mg	1.19	1.80	1.64	1.54
L-Glutamic acid	g	3.55	5.80	7.64	5.66
Glycine	g	4.52	7.20	5.93	5.88
L-Histidine	g	1.81	2.80	3.30	2.64
L-Isoleucine	g	3.13	4.00	5.10	4.08

L-Leucine	g	4.87	6.80	8.70	6.79
L-Lysine	g	3.24	4.40	5.90	4.51
L-Methionine	g	0.83	1.30	1.40	1.18
L-Phenylalanine	mg	0.00	0.00	0.00	0.00
L-Proline	g	3.27	4.80	6.36	4.81
L-Serine	g	2.01	4.30	3.43	3.25
Taurine	mg	70.00	63.00	344.00	159.00
L-Threonine	g	3.13	4.80	3.29	3.74
L-Tryptophan	g	0.97	1.30	1.36	1.21
L-Tyrosine	g	4.58	6.00	5.93	5.50
L-Valine	g	3.61	4.30	4.36	4.09
L-Carnitine	mg	45.00	56.00	46.00	49.00

Content per 100 g	Unit	MEDIFOOD	MAMOXI	NUTRICIA	MEAN OF 3 L-AAs PS
Vitamin A	μg RE	0.00	877.00	1020.00	632.33
Vitamin C	mg	0.00	81.00	131.00	70.67
Vitamin D	μg	0.00	31.00	28.60	19.87
Vitamin E	mg α-TE	0.00	22.00	11.40	11.13
Vitamin K	μg	0.00	81.00	88.90	56.63
Vitamin B1. Thiamin	mg	0.00	2.00	1.52	1.17
Vitamin B2. Riboflavin	mg	0.00	3.00	1.80	1.60
Vitamin B3. Niacin	mg	0.00	26.00	25.40	17.13
Niacin (NE)	mg	0.00	48.00	ND	24.00
Vitamin B6	mg	0.00	2.00	2.06	1.35
Total folate	μg	0.00	157.00	359.00	172.00
Vitamin B12	μg	0.00	6.00	6.43	4.14
Pantotenic acid	mg	0.00	6.00	7.13	4.38
Biotin	μg	0.00	31.00	191.00	74.00
Choline	mg	0.00	157.00	545.00	234.00
Calcium	mg	0.00	1960.00	1320	1093.33

Chromium	μg	0.00	63.00	38.00	33.67
Copper	mg	0.00	2.60	1.90	1.50
Iodine	μg	0.00	313.00	209.00	174.00
Iron	mg	0.00	28.00	18.90	15.63
Magnesium	mg	0.00	313.00	382.00	231.67
Manganese	mg	0.00	6.00	1.90	2.63
Molybdenum	μg	0.00	138.00	89.40	75.80
Phosphorus	mg	0.00	833.00	1000.00	611.00
Selenium	μg	0.00	75.00	95.50	56.83
Zinc	mg	0.00	23.00	13.90	12.30
Potassium	mg	0.00	0.00	358.00	119.33
Sodium	mg	0.00	0.00	<60	0.00*
Chloride	mg	0.00	0.00	<50	0.00*
Fluoride	mg	0.00	1.00	ND	0.50
Myo-inositol	mg	0.00	294.00	ND	147.00
Salt	g	0.48	0.00	<0.15	0.24*

Explanation of L-AAs protein substitutes – powdered.

MEDIFOOD AFENIL BUDDY. Recommended serving of 22 g.

MAMOXI XPHE SMART K. Recommended serving of 8 g.

NUTRICIA PKU LOPHLEX NEUTRAL. Recommended serving of 28 g.

*Mean calculated on two or one product due to lack of data.
